# A Clinical Review of the Psychiatric Sequelae of Primary Hyperparathyroidism

**DOI:** 10.7759/cureus.19078

**Published:** 2021-10-27

**Authors:** Ryan Serdenes, Morgan Lewis, Seetha Chandrasekhara

**Affiliations:** 1 Psychiatry, Temple University Hospital, Philadelphia, USA; 2 Psychiatry, Lewis Katz School of Medicine at Temple University, Philadelphia, USA

**Keywords:** consultation liaison psychiatry, behavioral endocrinology, psychosomatic psychiatry, adult primary hyperparathyroidism, clinical neuroscience

## Abstract

Despite one-quarter of patients with primary hyperparathyroidism (PHPT) experiencing psychiatric symptoms, there remains a dearth of literature regarding the diagnosis and further management of psychiatric sequelae in PHPT. We aim to review the literature pertaining to the epidemiology, disease presentation, pathophysiology, diagnostics, and therapeutics regarding psychiatric sequelae of PHPT with an emphasis on clinical pearls for practicing psychiatrists. A literature search was conducted using the US National Library of Medicine’s PubMed resource using the following keywords in various combinations: primary hyperparathyroidism, neuropsychiatric, calcium, psychosis, mania, depression, catatonia, delirium, parathyroidectomy, and psychotropic medication. We discuss in depth all aspects of the diagnosis and management of psychiatric sequela in PHPT. We have also identified epidemiological trends, discussed the most common clinical presentations, and postulated possible mechanisms for psychiatric symptoms in PHPT. Psychiatrists should maintain diagnostic suspicion for PHPT in older adult female patients presenting with new-onset psychiatric illness. Several mechanisms involving the following may explain the variety of psychiatric symptoms in PHPT: tyrosine hydroxylase, parathyroid hormone, interleukin-6, monoamine oxidase, calcium, and the sodium-potassium adenosine triphosphatase transporter. We recommend psychiatrists take a symptom-oriented approach to management. Treating a patient’s psychosis, mania, depression, catatonia, delirium, or eating disorder pathology via conventional therapeutics seems like a rational approach despite the underlying medical etiology. Only parathyroidectomy has been proven to be definitive in the complete amelioration of psychiatric symptoms.

## Introduction and background

Primary hyperparathyroidism (PHPT) is a disease of autonomous excessive secretion of parathyroid hormone (PTH) from pathologic parathyroid glands. It is the third most common endocrinological disorder and costs the United States healthcare system hundreds of million dollars annually [1-2]. Unfortunately, PHPT remains considerably under-diagnosed as only one-third of patients with hypercalcemia are evaluated with a PTH level [3]. Additionally, despite one-quarter of patients with PHPT experiencing psychiatric symptoms, there remains a dearth of literature regarding the diagnosis and further management of psychiatric sequelae in PHPT [4]. To our knowledge, at the time of writing, the majority of psychiatric literature on PHPT is several decades old and comprises mostly case reports. Moreover, none of the published review articles comprehensively address the pathophysiology of PHPT-mediated psychiatric disease or evaluate practical concerns in both diagnosis and psychiatric symptom management. This is surprising considering the seemingly ubiquitous education of PHPT-related symptoms in medical schools via the mnemonic “Stones, Bones, Groans, Moans,” with the “moan” component referring to psychiatric manifestations. Hence, herein we aim to be the first to comprehensively synthesize and review the collective literature pertaining to the epidemiology, disease presentation, pathophysiology, diagnostics, and therapeutics regarding psychiatric sequelae of PHPT with an emphasis on clinical pearls for practicing psychiatrists. Our hope is that this review will effectively fill knowledge gaps for curious clinicians as well as provide intellectual scaffolding for future systematic and scientific investigations.

## Review

Methods

**Figure 1:**
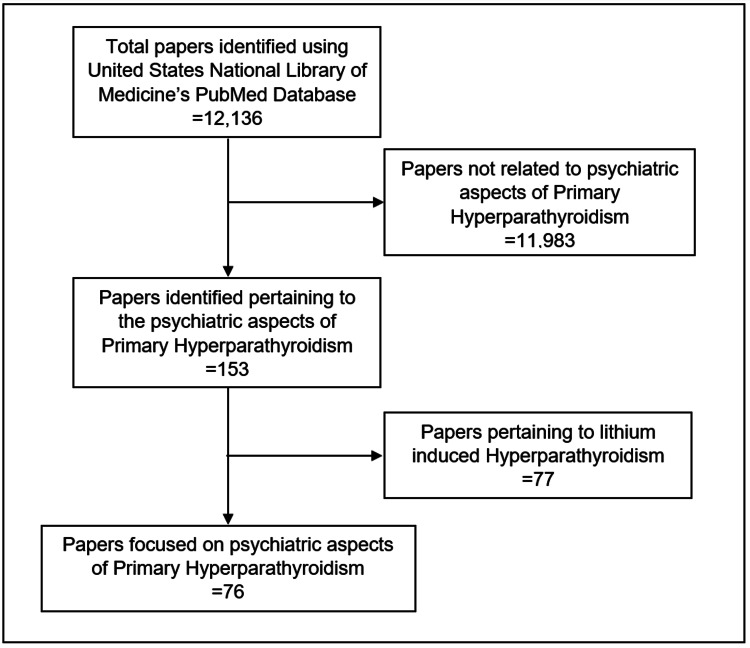
Consort diagram depicting our identification of papers focusing on psychiatric aspects of primary hyperparathyroidism

We reviewed papers on the psychiatric diagnosis and management of PHPT. A literature search was conducted in April 2021 using the US National Library of Medicine’s PubMed.gov resource (https://www.ncbi.nlm.nih.gov/pubmed). The following keywords in various combinations were searched: primary hyperparathyroidism, neuropsychiatric, calcium, psychosis, mania, depression, catatonia, delirium, parathyroidectomy, and psychotropic medication. Additional studies were identified by examining the reference lists of searched articles. Due to the relative lack of controlled studies, we included case reports. We excluded papers related to lithium-induced hyperparathyroidism as it has a distinct pathophysiology and we sought to limit confounding variables [5]. A consort diagram depicting our literature search can be found in Figure 1. Of note, this review did not utilize the Preferred Reporting Items for Systematic Reviews and Meta-Analyses (PRISMA) because of the limitations of the existing literature within this subject. As a result, we focused primarily on describing and synthesizing major findings as opposed to formally evaluating literature quality with an evidence-based rating scale. For comprehensiveness, especially regarding the non-psychiatric components of our review, we sourced literature from medical and basic science journals.

Discussion

Epidemiology

PHPT is the third most common endocrinological disorder behind thyroid disease and diabetes mellitus [6]. It has an estimated prevalence of 0.86% and is the most common cause of hypercalcemia [3,7]. In fact, PHPT is the underlying etiology of hypercalcemia in approximately 43% of patients [3]. There are notable trends within PHPT incidence and prevalence data that provide insights into disease risk factors and highlight specific patient populations in which clinicians should maintain high diagnostic suspicion.

Prior research has established associations of biological sex, age, and race with the incidence of PHPT. In the largest epidemiological study to date, Yeh et al. reported an age-dependent phenomenon where incidence is similar across sexes among those <50 years old (~12-24 per 100,000 person-years) but diverges when examining patients >50 years old. While they found the incidence of PHPT to increase in both sexes with increasing age, this increase is more substantial in females. In fact, for patients in the eighth decade of life, the incidence of PHPT was found to be approximately 95 and 196 per 100,000 person-years for males and females, respectively. Additionally, among racial groups, Blacks were noted to have significantly increased incidence and prevalence when compared to Whites, Asians, and Hispanics [8]. Considering this, psychiatrists should maintain diagnostic suspicion for PHPT in geriatric Black female patients presenting with psychiatric complaints.

Interestingly, while the pathophysiological underpinnings of race-associated differences in PHPT epidemiology remain uncertain, there have been several postulates within the literature attempting to ascertain the mechanistic relationship between patient age and incidence of PHPT. From a diagnostic perspective, increased osteoporosis screening among older adults has been identified as a contributor to age-dependent increases in incidence data [9]. Furthermore, it is important to note that age-related changes in PHPT incidence are not associated with renal impairment or vitamin D imbalance. Rather, complex estrogen-mediated mechanisms have been implicated, which may explain an increased incidence in females [10-12]. There have not been any identified psychiatric diseases known to us that confer a higher risk of PHPT. However, this may be a result of limited research.

Clinical Presentation

Psychiatric manifestations of PHPT are variable and can include the following symptomatology: depression, mania, psychosis, catatonia, and delirium [4,13,14]. Most case reports document symptom onset over the course of weeks to months. There have been several studies evaluating the commonality of different psychiatric symptoms in PHPT; however, the majority have small sample sizes and ill-defined methodology. The largest and most robust study into PHPT psychiatric symptomatology was by Joborn et al. that included a total of 441 patients. They found that approximately a quarter of the patients they evaluated with PHPT exhibited psychiatric symptoms. Consistent with epidemiological data, the majority of these patients were in their eighth decade of life. The most common reported psychiatric symptoms were fatigue, memory impairment, concentration difficulties, sadness, anxiety, and insomnia. Depressive states were found to manifest in around 75% of patients, while delirium and psychosis were less frequent at around 20% and 3%, respectively [4]. Mania and catatonia have only been documented in a few case studies of patients with PHPT, so it is likely they are less common than the aforementioned symptoms [13,14] Even rarer are presentations of eating disorders, as only one case has been reported within the literature [15]. To our knowledge, there has not been any documented presentations of PHTP manifesting with obsessive-compulsive and related disorders, trauma and stressor-related disorders, impulse control disorders, paraphilic disorders, or personality disorders. Considering the limitations of existing research, it is possible the frequency of various psychiatric symptoms differs in clinical practice from what we described herein. While depressive presentations are most likely, given the heterogeneity of psychiatric symptomatology in PHPT, psychiatrists should entertain the possibility of a diagnosis in alternative clinical scenarios - especially in older adult patients presenting with new-onset psychiatric illness.

Pathophysiology

PHPT most commonly results from sporadic adenomas in approximately 85% of cases. Alternative pathologies such as hyperplasia and multiple adenomas are less likely, with parathyroid carcinoma being the rarest etiology. Despite numerous identified genetic risk factors for the disorder, the majority of patients with PHPT are without a family history of parathyroid disease or other endocrine pathology [16]. When considering the underlying pathophysiology of psychiatric symptoms in PHPT, it is likely the mechanistic underpinnings are multifactorial - especially given the substantial diversity of clinical presentations. While several publications have offered postulates on how different components of PHPT can contribute to mental illness, none to our knowledge have developed a cohesive theoretical framework explaining the multitude of psychiatric presentations from a single disease entity [17-19]. However, when evaluating the clinical literature alongside the basic science research, a hypothetical model explaining PHPT-induced psychiatric symptoms can be synthesized.

**Figure 2:**
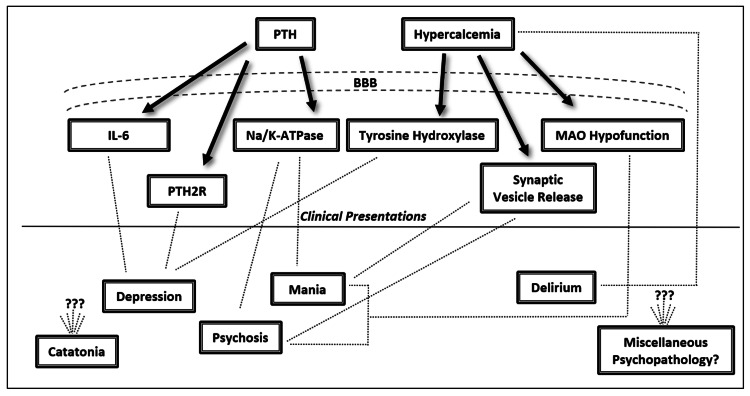
Proposed pathophysiologic correlates of psychiatric symptomatology in primary hyperparathyroidism BBB = blood brain barrier, PTH = parathyroid hormone, IL-6 = interleukin-6, Na/K-ATPase = sodium-potassium adenosine triphosphatase transporter, MAO = monoamine oxidase, PTH2R = PTH2 receptors, ??? = unknown mechanisms

The majority of research into PHPT-induced psychiatric symptoms converges on a monoamine hypothesis [18,19]. Generally, when considering the available research, two pathophysiological themes emerge centered on effects of PHPT that either increase or decrease monoamine neurotransmission. The combination of these components along with each patient’s unique neural architecture is likely sufficient to explain the variability of clinical presentations. In our review of the literature, we have identified six total variables: tyrosine hydroxylase, PTH, interleukin-6 (IL-6), monoamine oxidase (MAO), calcium, and the sodium-potassium adenosine triphosphatase transporter (Na/K-ATPase). See Figure 2 for the proposed pathophysiological processes underlying psychiatric symptoms in PHPT.

Biological factors increasing monoamine neurotransmission include decreased MAO function, decreased Na/K-ATPase function, and increased synaptic calcium. Hypercalcemic states have been implicated in impairing MAO enzymatic activity via reducing substrate affinity and consequently allowing for a theoretical increase in the transmission of monoamines such as serotonin, norepinephrine, and dopamine [20]. Additionally, via unknown mechanisms, PTH has been demonstrated to inhibit Na/K-ATPase-dependent reuptake of synaptic norepinephrine resulting in increased concentrations within the synaptic cleft [21]. Finally, hypercalcemia is suggested to increase monoamine neurotransmission via presynaptic calcium influx and subsequent calcium- dependent synaptic vesicle release [13].

Biological factors decreasing monoamine neurotransmission include decreased tyrosine hydroxylase function, increased cerebral PTH, and increased IL-6 levels. Tyrosine hydroxylase function has been found to be impaired in hypercalcemic states via unknown mechanisms and leads to decreased levels of norepinephrine and dopamine [20]. With regard to PTH, it has been demonstrated to cross the blood brain barrier and be associated with depressed states independent of hypercalcemia or calciferol [22-24]. Also, since PTH is a ligand for PTH2 receptors localized to the limbic system, this may be a pathway for its psychiatric effects [25]. PTH-dependent neuropsychiatric effects may also be mediated by monoamines given altered levels of cerebrospinal fluid 5-hydroxyindoleacetic acid and homovanillic acid found in hyperparathyroidism [19]. Additionally, PTH may be implicated with increases in cytokines such as IL-6 that can mediate monoamine neurotransmission and has been associated with depression [26, 27].

Considering the aforementioned processes, it is possible to speculate that PHPT-mediated elevations in monoamine neurotransmission may be responsible for psychotic and manic presentations. Conversely, PHPT-mediated decreases in monoamine neurotransmission may be responsible for depressed presentations. In addition to being influenced by aberrant monoamine neurotransmission, PHPT-related delirium may also be influenced by calcium levels, as concentrations above >13.5 mg/dL have been demonstrated to cause stupor [28]. While catatonia and eating disorders have complex neurobiology, both have monoamines implicated in their pathophysiology which can be affected in PHPT [15,29]. Aside from monoamine modulation, PHPT may have alternative mechanisms of inducing psychiatric symptoms since calcium is a secondary messenger and can influence a variety of neurobiological processes [30]. Calcium may even have concentration-dependent effects on psychiatric manifestations; however, there is alternative literature disputing this [4,19]. Regardless of the exact pathophysiology, there are multiple neurobiological processes sufficient to explain the diversity of psychiatric pathology found in PHPT.

Diagnosis

**Figure 3:**
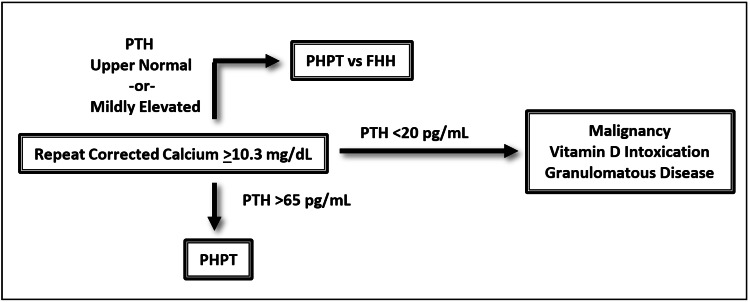
General diagnostic schema for hypercalcemia Of note, this does not account for clinical scenarios where ionized calcium measurements may be indicated, for cases of early disease, or normocalcemic variants of primary hyperparathyroidism. PHPT = primary hyperparathyroidism, PTH = parathyroid hormone, FHH = familial hypocalciuric hypercalcemia

From a medical symptom perspective, complaints of fatigue, weakness, back pain, and joint pain most commonly present in over 50% of cases. Other frequent medical symptoms include polyuria, constipation, and pruritus. Furthermore, diagnostic suspicion should be maintained in patients with nephrolithiasis, bone fractures, gastric ulcers, hypertension, gout, and pancreatitis [31]. Obtaining a serum calcium is the first step in the evaluation of possible PHPT. Levels above 10.3 mg/dL should be repeated and corrected for albumin before conferring a diagnosis of hypercalcemia. There is a considerable amount of literature discussing the utility of measuring ionized calcium as it is more sensitive than serum calcium. However, serum calcium remains a reasonable first line assessment [32]. Determining PTH levels is the next step and levels above 65 pg/mL confirm the diagnosis of PHPT. PTH levels bordering the upper limit of normal can still be diagnostic of PHPT, but in these cases, the possibility of familial hypocalciuric hypercalcemia should be considered and additional diagnostics should be completed. Low serum levels of PTH (<20 pg/mL) necessitate additional workup for other pathology including malignancy, vitamin D intoxication, and chronic granulomatous disease [33-35]. In patients with high clinical suspicion of PHPT, there may be an indication to obtain a PTH level in the absence of hypercalcemia, as this can represent early disease or a normocalcemic variant of PHPT [36]. Of note, PHPT is a biochemical diagnosis and does not require imaging. Once the diagnosis of PHPT is made, preoperative localization studies such as ultrasound or sestamibi scan can then be utilized in preparation for surgical resection [37]. PHPT remains under-diagnosed as two-thirds of hypercalcemic patients will never be evaluated for elevated PTH [3]. Therefore, completing the aforementioned workup in patients with hypercalcemia is essential to their psychiatric care, especially when it remains unlikely that a full evaluation will be completed by alternative providers. See Figure 3 for a diagnostic schematic for hypercalcemia.

Management

The medical management of PHPT involves both pharmacologic and nonpharmacologic strategies. Generally, it is recommended to encourage adequate hydration to minimize the risk of nephrolithiasis, physical activity to reduce bone resorption, and maintaining intake of Vitamin D and calcium, as diminished consumption can further exacerbate aberrant PTH secretion. Avoiding hypercalcemia-inducing medications such as thiazide diuretics and lithium is also advisable. Pharmacologic interventions include bisphosphonates or calcimimetic monotherapy. In treatment refractory patients, combination therapy with both a bisphosophonate and calcimimetic can be employed. Estrogens and selective estrogen receptor modulators can also be used as therapeutics; however, these hormonal treatments have substantial risks and should only be utilized as last resort pharmacotherapy [34]. It remains unclear the degree of effect these treatments have on the psychiatric elements of PHPT.

Unfortunately, there is a scarcity of literature surrounding the psychopharmacologic management of patients with psychiatric manifestations of PHPT. Pimozide and verapamil have been proposed by several publications as they both have central calcium channel blocking properties [38]. By this logic, psychotropics such as gabapentin and lamotrigine may also have efficacy given their modulatory effects on calcium channels [39,40]. However, at the time of writing, there have been no randomized controlled trials evaluating the efficacy of various psychotropics or official consensus guidelines in the psychiatric management of PHPT. Therefore, we recommend psychiatrists take a symptom-oriented approach to management. Treating a patient’s psychosis, mania, depression, catatonia, delirium, or eating disorder pathology via conventional therapeutics seems like a rational approach despite the underlying medical etiology. Limiting the use of lithium, if possible, would be advisable given its propensity to exacerbate hyperparathyroidism [41]. Implementing theory-based treatments such as pimozide, verapamil, lamotrigine, or gabapentin into management may be appealing, but should be done only if the patient’s symptomatology permits. Of course, providers should expect reduced treatment responses to psychopharmacological interventions given that only a parathyroidectomy has been proven to fully ameliorate psychiatric symptoms in PHPT.

Surgical care of patients with PHPT includes parathyroidectomy that is indicated for patients with symptomatic disease. Historically, the definition of symptomatic disease was limited to symptomatic hypercalcemia, nephrolithiasis, and bone fractures [34]. The 2014 International Workshop guidelines for asymptomatic disease expanded the indications for parathyroidectomy by including additional parameters such as presence of vertebral fracture, a significant reduction in bone mineral density, creatinine clearance <60 cc/min, or an increase in serum calcium of >1 mg/dL above the upper limit of normal. Of note, neuropsychiatric symptoms were left out as a surgical indication despite their ubiquity and significant burden on patients [42]. Since then, several studies have recognized the efficacy of parathyroidectomy in ameliorating PHPT-induced psychiatric symptoms [26,41]. In a large prospective study, Liu et al. demonstrated a significant reduction in depressive and anxious symptoms post-parathyroidectomy and recommended considering neuropsychiatric symptoms as a relative indication for surgical intervention [43]. Some publications estimate that psychiatric symptoms resolve over the course of weeks to months after parathyroidectomy [26]. This has been consistent with our own personal clinical experience. At this time, it is unclear if certain psychiatric symptoms are more likely to resolve with surgery. In summary, we recommend psychiatrists consider surgical intervention in patients with clinically significant psychiatric symptoms. Collaboration with our surgical and endocrinological colleagues when discussing the risks and benefits of parathyroidectomy is advisable.

Strengths and Limitations

Our thorough synthesis of the literature related to the epidemiology, presentation, pathophysiology, diagnostics, and therapeutics of psychiatric sequelae in PHPT is a major strength of our paper, especially considering there is no review with equivalent comprehensiveness currently known to us. Despite this, limitations of this review include the advanced age of many sources and that we did not formally evaluate literature quality with an evidence-based rating scale. Shortcomings in the quantity of recent research restricted our ability to include more contemporary papers and as a result this limitation would be present in any current review. Notably, only 17 of the 76 identified papers in our search were published within the last 10 years. Consequently, this limits the value of pursuing any formal scoring of literature rigorousness, especially considering case reports comprise a significant portion of the psychiatric literature within the subject.

## Conclusions

PHPT is an important component of the psychiatric differential diagnosis, especially in older adult females with new-onset mental illness. Clinicians should appreciate the heterogenic breadth of psychiatric presentations associated with PHPT and maintain high diagnostic suspicion for the illness, particularly in patients with hypercalcemia. Completing appropriate laboratory evaluations is essential, as the majority of PHPT remains undiagnosed. Parathyroidectomy remains the most effective therapeutic intervention for psychiatric symptoms associated with PHPT; however, perhaps targeted psychotropic interventions can be utilized instead of a symptom-oriented approach as disease pathophysiology is further elucidated. In the future, systemic measures to reduce under-diagnosis in psychiatric patients as well as obtaining more prognostic data on various treatment outcomes would be beneficial.

## References

[1] Fraser WD (2009). Hyperparathyroidism. Lancet.

[2] Melton LJ III (1991). Epidemiology of primary hyperparathyroidism. J Bone Miner Res.

[3] Press DM, Siperstein AE, Berber E (2013). The prevalence of undiagnosed and unrecognized primary hyperparathyroidism: a population-based analysis from the electronic medical record. Surgery.

[4] Joborn C, Hetta J, Johansson H, Rastad J, Agren H, Akerström G, Ljunghall S (1988). Psychiatric morbidity in primary hyperparathyroidism. World J Surg.

[5] Shapiro HI, Davis KA (2015). Hypercalcemia and "primary" hyperparathyroidism during lithium therapy. Am J Psychiatry.

[6] Regal M, Páramo C, Luna Cano R, Pérez Méndez LF, Sierra JM, Rodríguez I, García-Mayor RV (1999). Coexistence of primary hyperparathyroidism and thyroid disease. J Endocrinol Invest.

[7] Adami S, Marcocci C, Gatti D (2002). Epidemiology of primary hyperparathyroidism in Europe. J Bone Miner Res.

[8] Yeh MW, Ituarte PH, Zhou HC (2013). Incidence and prevalence of primary hyperparathyroidism in a racially mixed population. J Clin Endocrinol Metab.

[9] Griebeler ML, Kearns AE, Ryu E, Hathcock MA, Melton LJ III, Wermers RA (2015). Secular trends in the incidence of primary hyperparathyroidism over five decades (1965-2010). Bone.

[10] Haden ST, Brown EM, Hurwitz S, Scott J, El-Hajj Fuleihan G (2000). The effects of age and gender on parathyroid hormone dynamics. Clin Endocrinol (Oxf).

[11] Khosla S, Atkinson EJ, Melton LJ III, Riggs BL (1997). Effects of age and estrogen status on serum parathyroid hormone levels and biochemical markers of bone turnover in women: a population-based study. J Clin Endocrinol Metab.

[12] Carrillo-López N, Román-García P, Rodríguez-Rebollar A, Fernández-Martín JL, Naves-Díaz M, Cannata-Andía JB (2009). Indirect regulation of PTH by estrogens may require FGF23. J Am Soc Nephrol.

[13] Brown SW, Vyas BV, Spiegel DR (2007). Mania in a case of hyperparathyroidism. Psychosomatics.

[14] Hockaday TD, Keynes WM, McKenzie JK (1996). Catatonic stupor in elderly woman with hyperparathyroidism. Br Med J.

[15] Ozawa Y, Koyano H, Akama T (1999). Complete recovery from intractable bulimia nervosa by the surgical cure of primary hyperparathyroidism. Int J Eat Disord.

[16] Carlson D (2010). Parathyroid pathology: hyperparathyroidism and parathyroid tumors. Arch Pathol Lab Med.

[17] Brown GG, Preisman RC, Kleerekoper M (1987). Neurobehavioral symptoms in mild primary hyperparathyroidism: related to hypercalcemia but not improved by parathyroidectomy. Henry Ford Hosp Med J.

[18] Joborn C, Hetta J, Rastad J, Agren H, Akerström G, Ljunghall S (1988). Psychiatric symptoms and cerebrospinal fluid monoamine metabolites in primary hyperparathyroidism. Biol Psychiatry.

[19] Joborn C, Hetta J, Niklasson F (1991). Cerebrospinal fluid calcium, parathyroid hormone, and monoamine and purine metabolites and the blood-brain barrier function in primary hyperparathyroidism. Psychoneuroendocrinology.

[20] Islam A, Smorgorzewski M, Zayed MA, Massry SG (1992). Effect of chronic renal failure with and without secondary hyperparathyroidism on the activities of synaptosomal tyrosine hydroxylase and monoamine oxidase. Nephron.

[21] Smogorzewski M, Campese VM, Massry SG (1989). Abnormal norepinephrine uptake and release in brain synaptosomes in chronic renal failure. Kidney Int.

[22] Harvey S, Hayer S, Sloley BD (1993). Parathyroid hormone-induced dopamine turnover in the rat medial basal hypothalamus. Peptides.

[23] Cengiz K, Ozkan A (1998). Depression and secondary hyperparathyroidism in chronic renal failure. Nephron.

[24] Hoogendijk WJ, Lips P, Dik MG, Deeg DJ, Beekman AT, Penninx BW (2008). Depression is associated with decreased 25-hydroxyvitamin D and increased parathyroid hormone levels in older adults. Arch Gen Psychiatry.

[25] Roman SA, Sosa JA, Pietrzak RH, Snyder PJ, Thomas DC, Udelsman R, Mayes L (2011). The effects of serum calcium and parathyroid hormone changes on psychological and cognitive function in patients undergoing parathyroidectomy for primary hyperparathyroidism. Ann Surg.

[26] Chiba Y, Satoh K, Ueda S, Kanazawa N, Tamura Y, Horiuchi T (2007). Marked improvement of psychiatric symptoms after parathyroidectomy in elderly primary hyperparathyroidism. Endocr J.

[27] Zalcman S, Murray L, Dyck DG, Greenberg AH, Nance DM (1998). Interleukin-2 and -6 induce behavioral-activating effects in mice. Brain Res.

[28] Tonini G, Tatò L, Rigon F (2004). Hyperparathyroidism. Minerva Pediatr.

[29] Rasmussen SA, Mazurek MF, Rosebush PI (2016). Catatonia: our current understanding of its diagnosis, treatment and pathophysiology. World J Psychiatry.

[30] Dubovsky SL, Murphy J, Christiano J, Lee C (1992). The calcium second messenger system in bipolar disorders: data supporting new research directions. J Neuropsychiatry Clin Neurosci.

[31] Chan AK, Duh QY, Katz MH, Siperstein AE, Clark OH (1995). Clinical manifestations of primary hyperparathyroidism before and after parathyroidectomy. A case-control study. Ann Surg.

[32] Tee MC, Holmes DT, Wiseman SM (2013). Ionized vs serum calcium in the diagnosis and management of primary hyperparathyroidism: which is superior?. Am J Surg.

[33] Shane E (2020). Diagnostic approach to hypercalcemia. UpToDate.

[34] Silverberg S, Fuleihan GE (2019). Primary hyperparathyroidism: management. UpToDate.

[35] Fuleihan GE, Silverberg S (2020). Primary hyperparathyroidism: diagnosis, differential diagnosis, and evaluation. UpToDate.

[36] Cusano NE, Silverberg SJ, Bilezikian JP (2013). Normocalcemic primary hyperparathyroidism. J Clin Densitom.

[37] Yip L, Silverberg SJ, Fuleihan GE (2020). Preoperative localization for parathyroid surgery in patients with primary hyperparathyroidism. UpToDate.

[38] Alarcón RD, Franceschini JA (1984). Hyperparathyroidism and paranoid psychosis. Case report and review of the literature. Br J Psychiatry.

[39] Sutton KG, Snutch TP (2001). Gabapentin: a novel analgesic targeting voltage‐gated calcium channels. Drug Dev Res.

[40] Pisani A, Bonsi P, Martella G (2004). Intracellular calcium increase in epileptiform activity: modulation by levetiracetam and lamotrigine. Epilepsia.

[41] Parks KA, Parks CG, Onwuameze OE, Shrestha S (2017). Psychiatric complications of primary hyperparathyroidism and mild hypercalcemia. Am J Psychiatry.

[42] Bilezikian JP, Brandi ML, Eastell R, Silverberg SJ, Udelsman R, Marcocci C, Potts JT Jr (2014). Guidelines for the management of asymptomatic primary hyperparathyroidism: summary statement from the Fourth International Workshop. J Clin Endocrinol Metab.

[43] Liu JY, Peine BS, Mlaver E (2021). Neuropsychologic changes in primary hyperparathyroidism after parathyroidectomy from a dual-institution prospective study. Surgery.

